# A comprehensive survey of warfarin-induced hepatic toxicity using histopathological, biomarker, and molecular evaluation

**DOI:** 10.1016/j.heliyon.2024.e26484

**Published:** 2024-02-18

**Authors:** Mona M. Atia, Heba Allah Ahmed Mahmoud, Magdy Wilson, Elham A. Abd-Allah

**Affiliations:** a Laboratory of Molecular Cell Biology, Zoology Department, Faculty of Science, Assiut University, Egypt; b Plant Protection Research Institute (PPRI), Agriculture Research Center, Animal Pests Department, Egypt; c Laboratory of Physiology, Department of Zoology, Faculty of Science, New Valley University, EL-kharga, Egypt

**Keywords:** Oral warfarin intake, Hemorrhage, Oxidative stress, Liver damage, Fibrosis, Antioxidant

## Abstract

Warfarin finds human application as anticoagulant therapy. Warfarin usage can cause liver damage and hemorrhage. Besides functioning as anticoagulant and causing continuous bleeding of pests, the mechanism of toxicity of warfarin is unknown. In this study, Wild female and male rats were administrated orally with warfarin for 18 days at 9, 18, 27.5, and 55 mg/kg, respectively. Hepatoxicity was determined by assessing, LD50, leukocyte counts, immunochemistry, histopathology, serum proteins, Western blotting, especially of markers of liver injury, such as AST, ALT & ALP, and markers of antioxidant and oxidative stress markers. Warfarin treatment decreased Nrf2 levels while it increased caspase 3, CYP2C9, COLL1A1. It caused cellular damage and fibrosis of liver. The plasma levels of markers of liver injury, AST, ALT, ALP, bilirubin and transferrin were increased. The plasma levels of albumin, IgG and antitrypsin were decreased. Warfarin treatment decreased RBC and total lymphocyte count while increasing selectively neutrophils. Warfarin exposure caused increased oxidative stress; increased LPO and decreased GSH, SOD, CAT and NO production. Oral exposure of rats with Warfarin leads to increased oxidative stress resulting into liver damage via CYP2C9 mediated by Nrf2 depletion.

## Highlights

• Warfarin induces increment oxidative stress and suppresses immunosenescence in the wild rat.
• The bleeding that causes mortality in the wild rat are accelerated by warfarin's stimulation of CYP2C9 pathway.
• Warfarin promotes fibrosis and pathogenesis of acute liver injury by increasing hemosiderin, and lipid peroxidation.

## Graphical abstract

**Graphical abstract** shows warfarin increased oxidative stress, leading to toxicity in the livers of wild rats (female and male), by different mechanisms pathway.

**Figure:**
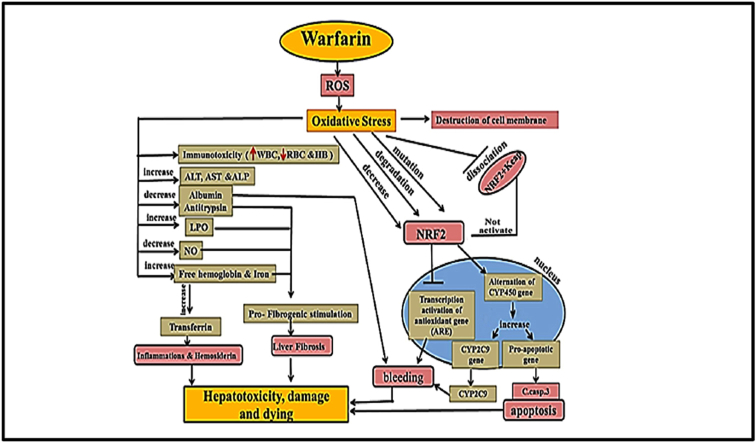

## 1 Introduction

Warfarin is a vitamin K antagonist, widely used as anticoagulant is currently used to treat individuals at high risk of blood clots [1,2]. Warfarin use is restricted since it can cause bleeding in cerebral, subcutaneous, and gastrointestinal when exposed to them [3]. The sodium salt of warfarin is being used as rodenticide in home, agriculture, commercial and industrial settings [4]. When the anticoagulant action of warfarin is very toxic, the bleeding might occur as a side effect [5].

Symptoms of warfarin toxicity include hemorrhages under the skin near the neck region, and animals administered high doses were dead after four days. Rats exposed to high doses also suffered from calcification of the elastic lamellae of the arteries and the aortic heart valves [6]; ovarian and adrenal vasodilation; cellular infiltration in the livers [7]; kidney hemorrhage, known as warfarin-related nephropathy; increased oxidative stress; NO synthesis decreased; and urinary tract bleeding [8] and caspases activation mediated apoptotic cell death, which activates pro-inflammatory pathways [9]. Severe hepatitis can be a side effect of coumarin therapy, so the drug's potential hepatotoxic effects must be taken into account when prescribing and when monitoring patients [10].

In animals models such as rats, cell death after intracerebral hemorrhage by apoptotic pathways [11]. In rodents, coumarin causes hepatic necrosis or increased plasma transaminase activity [12]. In the rat, acute coumarin-induced hepatotoxicity is associated with a decrease in the activity of hepatic cytochrome P-450 and associated mixed function oxidase enzymes; although none of the coumarin derivatives investigated caused any major liver damage [13]. Warfarin exposure decreases albumin levels and hypoalbuminemia leads to increase in the free fraction of warfarin [14]. Inflammatory mediators such as matrix metalloproteinases (MMPs) are activated by oxidative stress. The blood brain barrier (BBB) is disrupted as a result of injury, resulting into vascular permeability and bleeding [15]. Furthermore, Small intestinal hematoma was once a rare occurrence in anticoagulant-treated individuals [16,17]. Some experts predict that the incidence of small-bowel hematoma will rise when long-term warfarin treatment is increased [18].

Several antioxidant mechanisms are involved in cellular redox homeostasis [19,20]. The Nrf2/Keap1 pathway that modulates the expression levels of antioxidant and detoxification enzymes [21]. Warfarin has been linked to an increase in alkaline phosphatase in liver [22]. Exogenous warfarin dosages may influence superoxide dismutase (SOD) and catalase (CAT) activity differently, depending on the type of tissue, since warfarin has been shown to have pro-oxidant characteristics. Variations in the activity of the antioxidant enzymes CAT and SOD seen in tissues of warfarin-treated rats could be considered to be an indirect sign of the connection between oxidative activity and inflammation, and not as a result of a direct effect on SOD activity [23].

In this study we investigated the acute toxicity of warfarin on oxidative stress and the immune response, as reflected in the morphology, blood and liver of wild rats. We examined the essential molecular pathways affected by warfarin, by evaluating the levels of CYP2C9, C. casp.3, COL1A1, and NRF2, which induce bleeding, apoptosis and inflammation.

## 2 Materials and methods

### 2.1 Chemicals

Warfarin tablets 5 mg (Zentiva, Prague, Czech Republic) were obtained from a local pharmacy in Istanbul. EGTA, SDS-PAGE chemicals, nitrocellulose membranes, protease inhibitor, mouse (1:1000) anti-p450 IgG, anti-NRF2, anti-COLL1A1 and cleaved caspase-3, goat anti-β actin IgG, and mouse anti-goat IgG-HRP were purchased from Sigma-Aldrich (St. Louis, MO, USA). GSH, superoxide dismutase, epinephrine, and 5,5′-dithio-bis-(2- nitrobenzoic acid); DTNB were purchased from Sigma-Aldrich. The activities of GSH-Px, GSH-ST, glucose-6-phosphte dehydrogenase (G6PD), and CAT were determined using commercial kits (Bio-Diagnostic Company, Egypt). All other substances were obtained at highest analytical score possible from local sources.

### 2.2 Ethics approval

All experimental protocols were performed according to regulations set by the National Institutes of Health guidelines. The research techniques were examined and approved by the Assiut University Faculty of Medicine's medical ethics committee **(IRB no:17300533)**.

### 2.3 Experimental design

Sixty adult female and male wild rats of the species *Rattus rattus*, weighing 150–200 g, were taken alive from poultry farms in the Assiut valley for the experiment. They were housed in separate traps for one week at room temperature (25 °C), with a typical 12 h light/12 h dark cycle in the animal house of the Zoology Department, Faculty of Science, Assiut University. All animals were fed on tomatoes, soggy bread, and tap water. Trapped rats were divided into six groups of ten animals each, including both sexes. Group (I) was the male control, group II was the female control, groups III and IV were female rats treated orally with warfarin for 18 days 1/4 ${\text{LD}}_{50}$ and ${\text{LD}}_{50}$ at 9 and 18 mg/kg, respectively. Groups V and VI were male rats treated orally with warfarin for 18 days at 27.5 and 55 mg/kg, respectively. The rats were dissected and blood was gathered in non-heparinized tubes, and the livers were diced for further examination.

### 2.4 LD50 determination

The acute oral warfarin LD50 of rats was calculated using the “Probit analysis” technique described by Refs. [23,24]. The acute oral warfarin of rats was determined. Doses of warfarin were prepared as 60, 70, 80, and 90 mg/kg for males, and 15, 20, 30, and 40 mg/kg for females. Five adult rats of each sex, caged individually, were administered each dose, and the mortality and time to death were recorded up to four days after treatment. The values were calculated after 96 h by “Probit analysis”.

### 2.5 Western blotting

The liver was homogenized with RIPA lysis buffer, the tissue samples were chopped and briefly sonicated on ice, and the protein concentration was measured. Proteins (20 μg) were separated using SDS-PAGE and then transferred to a nitrocellulose membrane. Membranes were then washed with 5% skim milk in TBS. The solution was incubated with primary antibodies overnight at 4 °C and then with HRP-conjugated secondary antibodies for 1 h at 24 °C, as recommended by the manufacturers. The immunoreactive bands were visualized using a chemiluminescent substrate kit. The optical densities of the bands were quantified and normalized by corresponding -actin (1:5000) using ImageJ software [25].

### 2.6 Immunohistochemistry

The paraffin-embedded tissues were deparaffinized and rehydrated in a series of ethanol solutions (100%–70% ethanol). As previously described (Paxinos and Franklin, 2019). They were then incubated with antibody against cleaved caspase 3 (1:10), after which the sections were washed and stained with 3, 3′-diaminobenzidine (DAB) for 2–3 min before being counterstained with hematoxylin for 2–5 min.

### 2.7 Estimation of serum protein fractions

Serum samples were recovered from clotted blood after centrifugation at 5000 rpm for 5 min, and the concentration of total serum protein was calculated. Aliquots of 20 μg of serum proteins were subjected to SDS-PAGE, and the gel was stained with Coomassie blue before being destained using 40% methanol and 10% acetic acid. The serum proteins were blotted against molecular weight standards expressed on a semi-logarithmic scale to establish their identity. ImageJ software was used to calculate the optical density of each band.

### 2.8 Leukocyte counts

Blood serum was collected as previously described, and differential white blood cells were counted using a CBC analyzer machine, the veterinary Exigo Hematology Analyzer at the Clinical Pathology Laboratory in the Pathology Department, Faculty of Veterinary Medicine, Assiut University.

### 2.9 Determination of alanine aminotransferase (ALT), and aspartate aminotransferase (AST)

Specific kits (Boehringer Mannheim, Mannheim, Germany) were used to assess ALT and AST activity in blood plasma, following the manufacturer’s protocols [26].

### 2.10 Estimation of total bilirubin, direct bilirubin, alkaline phosphatase, glucose, albumin, and total protein

As described by Ref. [27] plasma total bilirubin was measured using a commercial kit (Spectrum Diagnostics Company, Egypt). Blood glucose levels were determined according to the GOD-PAP enzymatic colorimetric method [28], and ALP activity was measured according to the method of [29], using a commercial kit from Bio-Diagnostic (El-Bouchrieh, Egypt). Serum albumin was measured using kits, as described by Ref. [30]. The concentration of total protein in the plasma and tissue cytosols was evaluated using the method of [31].

### 2.11 Lipid peroxidation and nitric oxide assessments

According to Ref. [32], the liver's lipid peroxidation (LPO) was measured using the thiobarbituric acid reaction. The amount of nitrite in the tissue cytosols of the organs was used to calculate the concentration of nitric oxide by method of [33].

### 2.12 Superoxide dismutase, catalase, and activity reduced glutathione assessments

SOD activity was determined by its ability to prevent the oxidation of epinephrine using the method of [34]. CAT activity was measured in accordance with [35] description. The GSH concentration was calculated in accordance to Ref. [36].

### 2.13 Histological preparation, histopathology score and fibrosis percentage

Tiny sections of liver tissue were fixed in 10% neutral formalin (pH 7.2) for histopathological investigation. Hematoxylin, eosin, and Masson stains were used to stain paraffin sections with a thickness of 5 mm. Five parameters of liver histopathology were measured: fibrosis, inflammation, pyknosis, region of degeneration, and nuclear fragmentation [37]. The collagen content of liver slices stained with Masson's trichrome was determined using morphometric analysis to calculate the fibrosis percentage [38,39].

### 2.14 The statistical methods

The data are shown as mean ± SE from at least three independent experiments. Student's t-tests were used to compare the two groups. The study ANOVA was used in the statistical analysis, and the difference was declared significant at P < 0.001.

## 3 Results

### 3.1 Determination of ${\text{LD}}_{50}$ and body weight

**Fig. 1:**
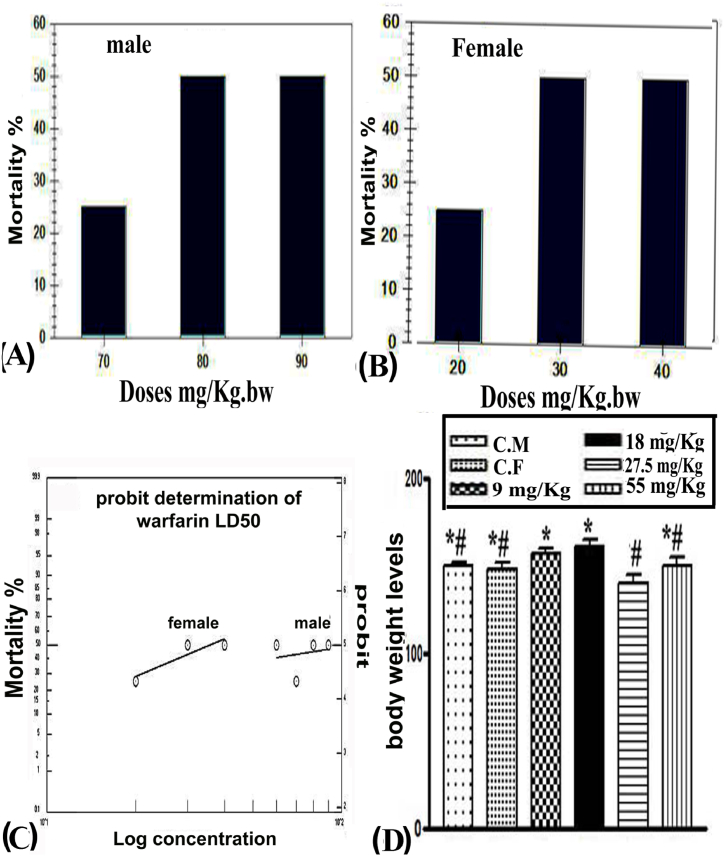
**(A–C)** Determination of LD50 in wild rats exposed to different oral doses of warfarin, **(D)** body weight. Data are expressed as mean ± SE. The values of different signs were significantly different (P < 0.001).

Oral doses of 60, 70, 80, and 90 mg/kg body weight were administered to male rats, and 15, 20, 30, and 40 mg/kg body weight were administered to female rats. The mortality was 0.0, 25, 50, 50, and 50% for males and females. Animals were found dead between days 4–6 after oral organization. The LD50 value for females was 35.97 mg/kg, lower than that for males, at 110.16 mg/kg (Fig. 1A–C).

The oral administration of warfarin for 18 days; the doses for female 9, 18 mg/kg and male 27.5 and 55 mg/kg non-significant decrease the body weight 6.4997, 9.4109, 6.7423, and 0.2670%, respectively of female and male wild rats versus those of control (Fig. 1D).

### 3.2 Pathomorphological changes in wild rats

**Fig. 2:**
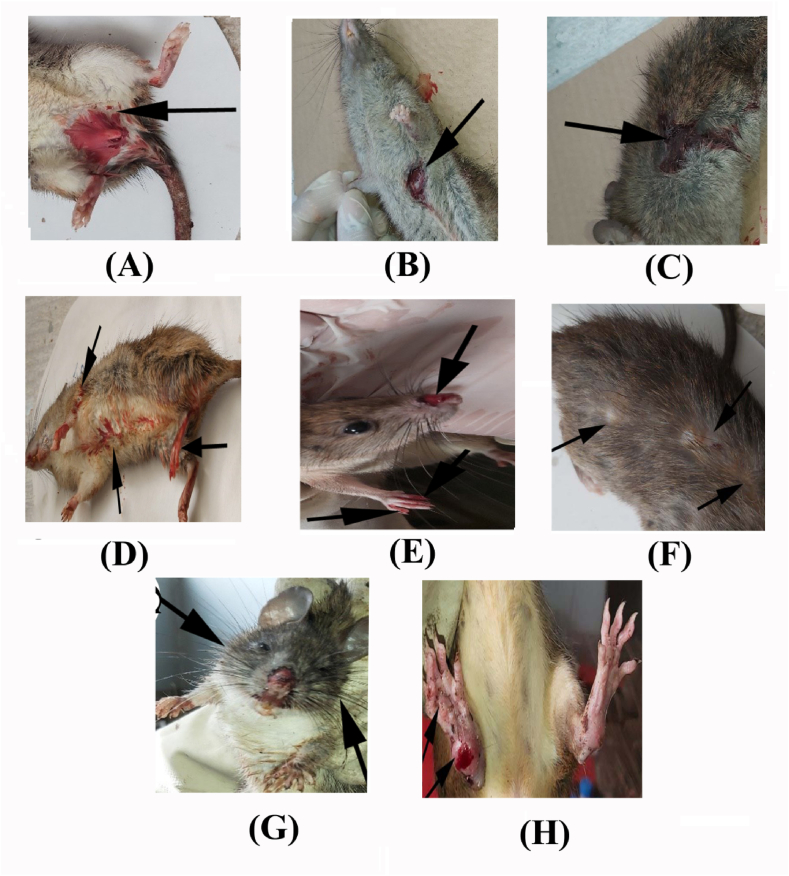
Pathomorphological symptoms in wild rats (male and female) after oral doses of warfarin (arrow): **(A, B, C, D, and E).** Bleeding in both sexes near the genital openings, abdomen, back, femur, nose, and the tips of the fingers. **(F)** Skin hair loss and alopecia. **(G)** Enlargement or swelled head. **(H)** Purple toe syndrome.

Warfarin toxicity symptoms appeared 2–4 days after oral administration, and become more severe 7 days later. Toxicity symptoms in animals included dormancy, emaciation, difficulty breathing, and hemorrhages in various regions including near the genital opening, the abdomen, back, femur, nose, eyes, and mouth, and the tips of the fingers and tail (Fig. 2A–E). Warfarin also induced alopecia, purple toe syndrome, and enlargement of the head in both sexes of rats (Fig. 2F–H).

### 3.3 Immunoblotting detection of P450 (CYP2C9), collagen 1 alpha 1 (COL1A1), and nuclear factor erythroid 2-related factor 2 (NRF2)

**Fig. 3:**
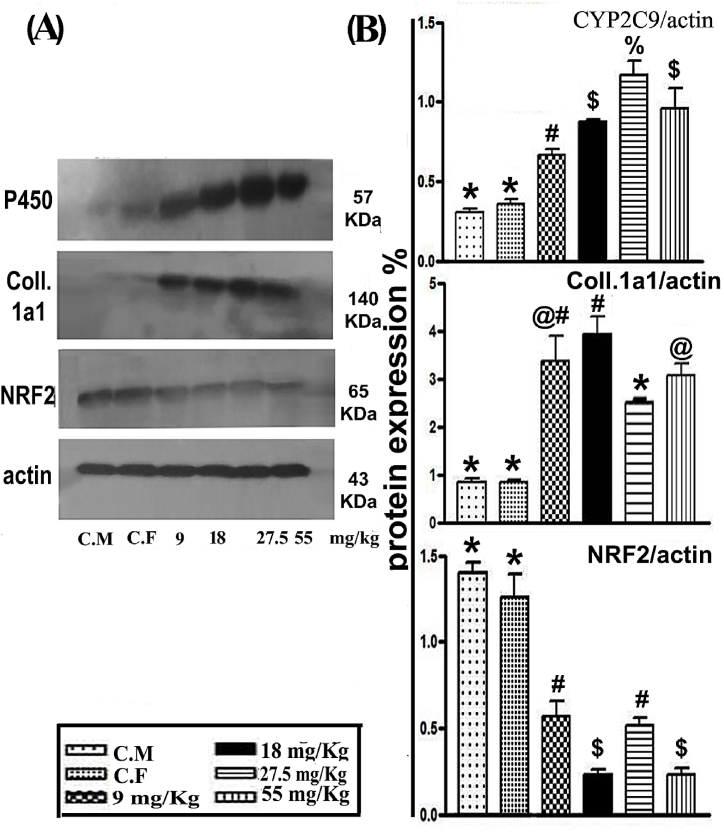
**(A)** Immunoblot detection showing changes in protein levels of CYP2C9, COL1a1, and NRF2 following different doses of warfarin. **(B)** Densitometric analysis. Qualities in the column with various signs are significantly different at P < 0.001.

Western blotting and densitometric estimation showed changes in the levels of CYP2C9, Coll.1A1, and NRF2 in the livers of female and male rats treated with doses of warfarin of 9, 18, 27.5, and 55 mg/kg. Female and male rats treated with warfarin at different doses had significantly increased levels of endogenous CYP2C9 and Coll.1A, of 1 85.2, 143.5, 285.5, and 216.1% in females, and 306.0, 382.3, 194.1, and 260.3% in males. In contrast, warfarin significantly decreased the levels of NRF2 by 55.0, 82.0, 63.1, and 83.3%, compared with control rats (Fig. 3A and B).

### 3.4 Stimulation of cleaved caspase 3 by warfarin

**Fig. 4:**
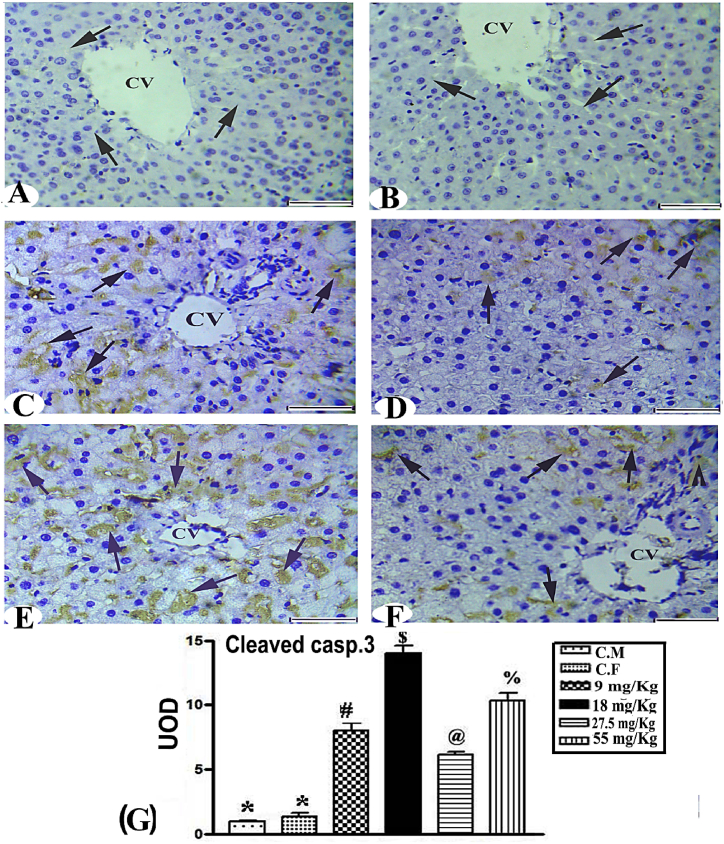
Immunohistochemical detection of C. caspase 3 of liver in female and male wild rats given different doses of warfarin, showing **(A and B)** control group negative reaction (arrow), **(C and E)** the 9 and 18 mg/kg groups showed strong positive reactions (arrows), **(D and F)** the 27.5 and 55 mg/kg groups had variable positive immunolabeled C. casp.3 (arrows) bar = 50 μm. **(G)** Statistical analysis, showing values of different signs were significantly different (P < 0.001).

Immunohistochemical detection of cleaved caspase 3 levels in the liver of female and male wild rats (Fig. 4A and B) indicated the disappearance of brown patches in the vast majority of hepatocytes of caspase 3 level in control liver in both sexes. (Fig. 4C and E). Rats administered 9 mg/kg warfarin had sharp increases in caspase 3, evident as homogenous brown patches in the cytoplasm. The localization of the brown patches in the 18 mg/kg group was variable (Fig. 4D and F) However, in the 27.5 and 55 mg/kg groups there was considerable aggregation of brown patches of caspase 3 (Fig. 4G). Warfarin at doses of 9 and 18 mg/kg in females had significant stimulatory effects of 383.77 and 532.8%, on caspase 3, which were higher than the effects of warfarin at doses of 27.5 and55 mg/kg for males, which increased caspase 3 by 207.9 and 329.8%, respectively.

### 3.5 Blood serum protein levels

**Fig. 5:**
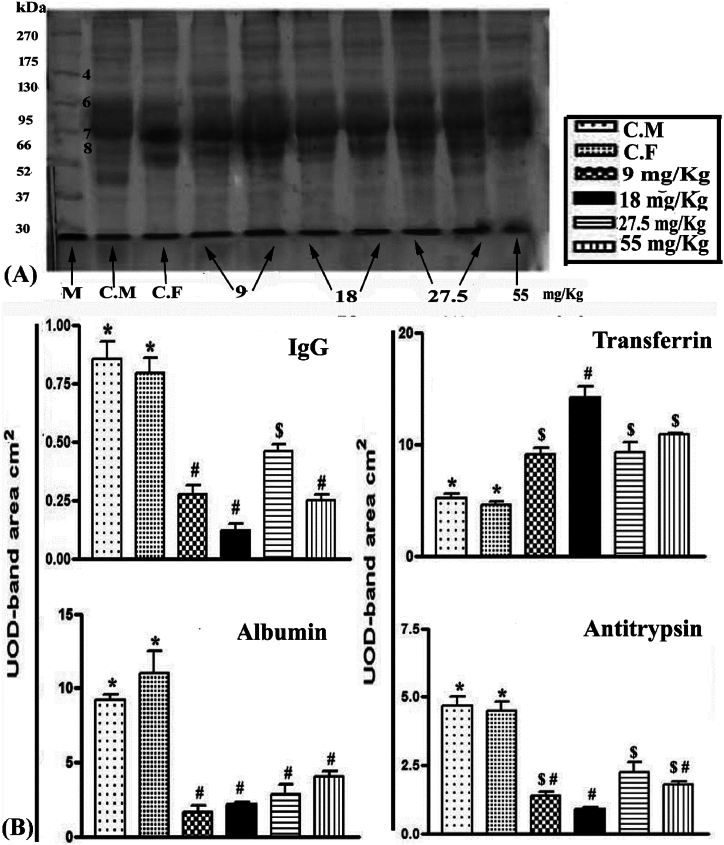
**(A)** SDS-PAGE of serum protein fractions of control and warfarin doses in wild rats, showing the patterns of major proteins: immunoglobulin G, transferrin, albumin, and antitrypsin (band 4, 168 KDa; band 6, 85 KDa; band 7, 64 KDa and band 8, 60 KDa),respectively. **(B)** Changes in the mean values of proteins levels with different sign were significantly different (P < 0.001).

The effects of warfarin administration on serum proteins were measured densitometrically by gel-layer electrophoresis. The proteins were immunoglobulin G heavy chain (IgG), transferrin, albumin, and antitrypsin as shown in Fig. 5A and B. Warfarin at doses of 9, 18, 27.5, and 55 mg/kg appeared to down-regulate the levels of IgG, albumin, and antitrypsin by 65.5, 84.9, 46.1, and 71%; 85.0, 80.4, 69, and 55.8%; and 69.4, 80.7, 52.2, and 62%, respectively. In contrast, it stimulated the transferrin level by 99.9, 212.6, 80.5, and 112.2%, respectively compared to control rats.

### 3.6 Assessment of CBC

**Fig. 6:**
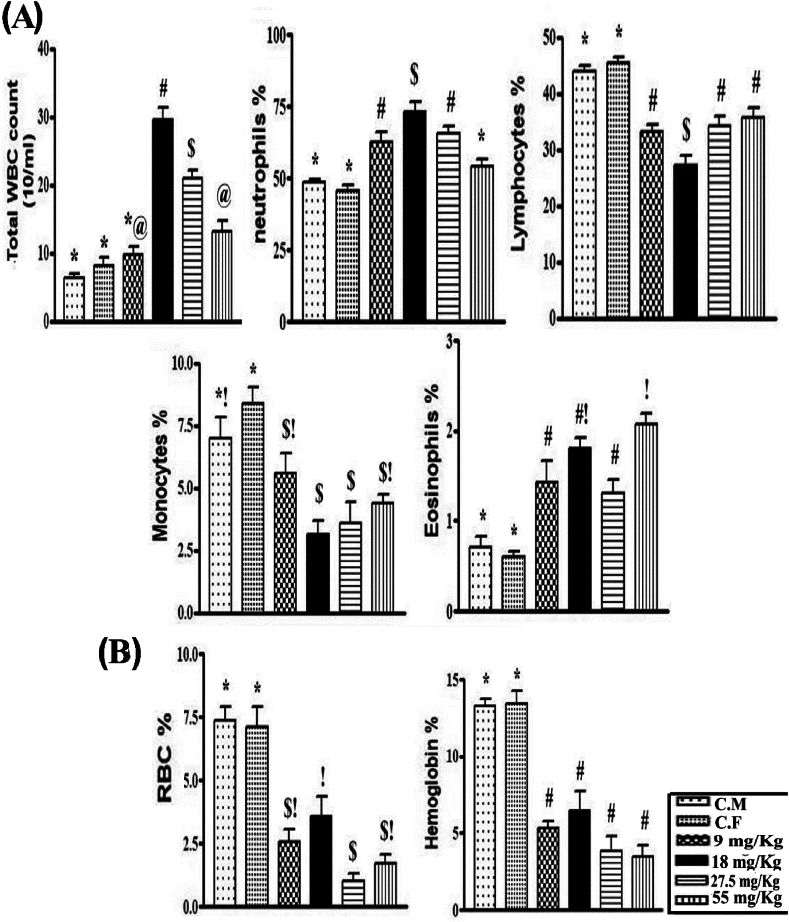
**(A and B)** White and red blood cells and hemoglobin percentage in control and warfarin-dosed wild rats. Data and values with various signs are presented as mean ± SE, and were significant at (P < 0.001).

Warfarin at doses of 9, 18, 27.5, and 55 mg/kg significantly (P < 0.001) increased the total white blood cell count, neutrophil, and eosinophil counts of wild rats by 19.5, 260.1, 226, and 103.6; 37, 60.5, 34.2, and 11; and 138.8, 200.0, 84.8, and 194 %, respectively. The warfarin experimental groups recorded significantly decreased lymphocytes and monocytes by 26.9, 40.0, 22.2, and 19.2; and 33.3, 62.7, 48.8, and 36.8%, respectively compared to control rats (Fig. 6 A). The percentage of red blood cells and hemoglobin decreased by 63.8, 49.8, 86.0, and 76.5576; and 60.4083, 52.2, 71, and 74% respectively, after oral administration of warfarin (Fig. 6B).

### 3.7 Biochemical parameters in plasma

The current work showed that AST levels in female rats treated with a dose of 0.25 LD_50_ and 0.5 LD_50_ were increased non-significantly, by 11.43% and 15.49%, respectively. The elevation in males treated with doses of 0.25 ${\text{LD}}_{50}$ and 0.5 LD_50_ were non-significant at 26.48% and significant at 47% respectively, compared with the controls (Table 1). ALT levels in females and males treated with doses of ¼ LD_50_ and ½ LD_50_ were decreased by 1.56% and increased by 5.59, 7.17, and 0.7% respectively; the elevation was non-significant. Total bilirubin level in female rats given a dose of ¼ LD_50_ were significantly decreased by 60.2%, and in those given ½ LD_50_ were increased by 54.1%. In males, the total bilirubin level following doses of ¼ ${\text{LD}}_{50}$ and ½ ${\text{LD}}_{50}$, were significantly increased by 177.2 and 106.1%, respectively. In the case of direct bilirubin, female rats treated with a dose ¼ LD_50_ and ½ LD_50_ were non-significantly decreased by 3.9% and significantly increased by 491.1%, respectively. In males treated with doses of ¼ LD_50_ and ½ ${\text{LD}}_{50}$ direct bilirubin was non-significantly increased by 217.82 and 117.2% respectively.

**Table 1:** Effect of Warfarin at different doses in some plasma activities of wild rats (***Rattus***).

	Male					Female				
	Control	27.5 mg/kg.b.w (1/4 LD_50_)		55 mg/kg.b.w (1/2 LD_50_)		Control	9 mg/kg.b.w (1/4 LD_50_)		18 mg/kg.b.w (1/2 LD_50_)	
	Mean ± SE	Mean ± SE	% of change	Mean& St.error	% of change	Mean ± SE	Mean ± SE	% of change	Mean& St.error	% of change
**AST (U/L)**	37.04 ± 4.027	46.85 ↑ ± 2.281	26.48 %	54.45 ↑ ± 1.457***	47 %	35.43 ± 0.926	39.48 ↑ ± 2.290	11.43 %	40.92 ↑ ± 3.559	15.49 %
**ALT (U/L)**	25.52 ± 1.192	27.35 ↑ ± 0.8578	7.17 %	25.70 ↑ ± 1.155	0.7 %	25.58 ± 0.711	25.18 ↓ ± 0.685	1.56 %	27.01 ↑ ± 0.8902	5.59 %
**Total Bilirubin (mg/dL)**	0.7821 ± 0.1414	2.168 ± ↑ 0.104***	177.2%	1.612 ↑ ± 0.147***	106.1%	1.216 ± 0.136	0.4837 ↓ ± 0.089**	60.2 %	1.874↑ ± 0.174**	54.1%
**Direct Bilirubin (mg/dL)**	0.7413 ± 0.328	2.356 ↑ ± 0.59	217.8 %	1.610 ↑ ± 0.508	117.2%	1.195 ± 0.5153	1.146 ↓ ± 0.312	3.9%	7.046 ↑ ± 0.36***	491.1%
**ALP Test**	8.43 ± 1.38	9.166 ↑ ± 0.4898	8.7%	25.42 ↑ ± 1.86***	201.5%	2.832 ± 0.4437	5.454 ↑ ± 2.83	92.6%	22.66 ↑ ± 1.26***	700.14%
**glucose (mg/L)**	159 ± 9.80	224.2 ↑ ± 8.91*	41%	178.8 ↑ ± 11.01	12.5%	231.6 ± 18.16	304.7 ↑ ± 23.3**	31.6%	247 ↑ ± 17	6.7%
**Albumin (mg/L)**	5.221 ± 0.343	2.997 ↓ ± 0.385*	42.6%	2.530 ↓ ± 0.477**	51.5%	3.754 ± 0.6243	3.512 ↓ ± 0.441	6.4%	3.060 ↓ ± 0.4938	18.5%
**Plasma total Protein (mg/ml)**	10.56 ± 0.131	6.977 ↓ ± 0.21***	33.92%	6.571 ↓ ± 0.139***	37.77%	11.34 ± 0.1899	8.961 ↓ ± 0.25***	20.97%	6.536 ↓ ± 0.10***	42.36%

Data are represented as mean ± SE. rats (n) = 5. *, ** and = *** significant difference from control at P < 0.05, P < 0.01, and P < 0.001 respectively.

The ALP levels of female and male rats treated with ¼ LD_50, and_ ½ LD_50_ were non-significantly and significantly respectively raised by 92.6, 700.15, 8.7, and 201.5%, respectively (Table 1). Changes in glucose values in females and males rats treated with ¼ LD_50_ and ½ LD_50_, were increased 31.6%, 6.7% (non-significant), and 41% and 12.5% (non-significant), respectively. Albumin levels in females and males treated with doses of ¼ LD_50_ and ½ LD_50_, were decreased non-significantly by 6.4, 18.5, 42.6, and 51.5%, respectively. Total protein levels in the plasma of females and males treated with doses of ¼ LD_50_ and ½ LD_50_ were significantly decreased by 20.97, 42.36, 33.92, and 37.77% respectively, all parameters compared with control groups (Table 1).

### 3.8 Antioxidants and oxidative stress biomarkers of liver

Hepatic total protein was significantly elevated in all treated groups, by 13.77, 76.74, 65.31, and 38.68% in female and male rats treated with doses of ¼ LD_50_ and ½ LD_50_, respectively (Table 2). GSH levels were non-significantly decreased in all treated groups. All of the treated groups showed significant decreases in SOD and CAT (Table 2). The oxidative stress biomarker LPO was significantly increased in the ¼ LD_50_ and ½ LD_50_ of female and male rats, by 145.4, 125, 37.41, and 77.45%, respectively. Finally, NO levels were significantly decreased in females and males at doses of ¼ LD_50_, and ½ LD_50_ by 29.09, 52.42, 10.63, and 62.03% respectively, compared to the control groups (Table 2).

**Table 2:** Effect of Warfarin at different doses on some antioxidants and oxidative stress biomarkers in liver of wild rats (***Rattus rattus***).

Parameters	Male					Female				
	Control	27.5 mg/kg.b.w		55 mg/kg.b.w		Control	9 mg/kg.b.w		18 mg/kg.b.w	
	Mean ± SE	Mean ± SE	% of change	Mean ± SE	% of change	Mean ± SE	Mean ± SE	% of change	Mean ± SE	% of change
**total Protein (mg/ml)**	3.804 ± 0.085	3.280↓ ± 0.077***	13.77%	0.885↓ ± 0.041***	76.74%	3.745 ± 0.07297	1.300↓ ± 0.090***	65.31%	2.298↓ ± 0.090***	38.68%
**GSH ng/mg protein**	4.947 ± 0.264	4.488↓ ± 0.492	9.27%	3.767↓ ± 0.227	23.85%	5.278 ± 0.2947	5.121↓ ± 0.2773	2.97%	4.258↓ ± 0.3772	19.32%
**SOD ng/mg protein**	8.506 ± 0.319	3.780↓ ± 0.502***	55.56 %	5.011↓ ± 0.193***	41.08%	6.133 ± 0.3277	4.683↓ ± 0.1976*	23.64%	3.780↓ ± 0.502***	37.04%
**CAT activity U/min/mg protein**	144.5 ± 7.360	105.9↓ ± 3.309***	26.24%	103.4↓ ± 3.618***	28.59%	173.9 ± 7.976	118.9↓ ± 9.034***	31.62%	102.2↓ ± 5.287***	41.23%
**LPO nmol/mg protein**	4.683 ± 0.419	6.435↑ ± 0.245**	37.41%	8.310↑ ± 0.446***	77.45%	3.392 ± 0.3548	8.324↑ ± 0.317***	145.4%	7.632↑ ± 0.415***	125%
**NO nmol/mg protein**	2.002 ± 0.148	1.789↓ ± 0.166***	10.63%	0.760↓ ± 0.101***	62.03%	2.396 ± 0.1152	1.699↓ ± 0.122**	29.09%	1.140↓ ± 0.115***	52.42%

Data are represented as mean ± SE. rats (n) = 5. *, ** and = *** significant difference from control at P < 0.05, P < 0.01, and P < 0.001 respectively.

### 3.9 Histological examination of liver

**Fig. 7:**
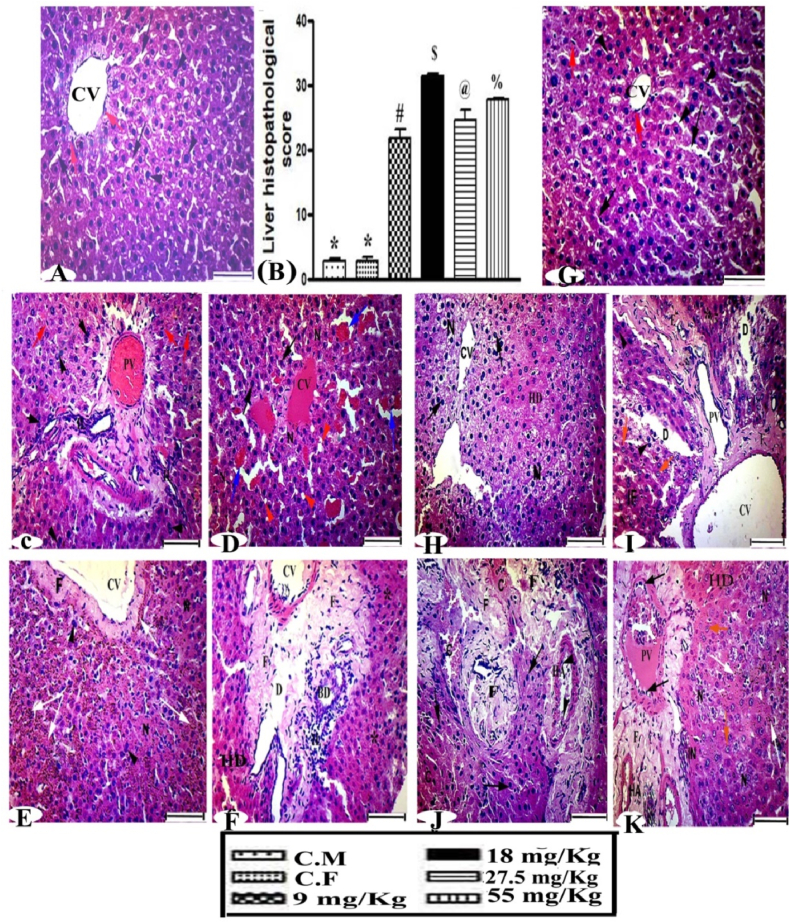
Liver examination of wild rats **shown (A and G**) control group with central vein (CV), hepatocytes (arrow head), and Kupffer cells (red arrow). **(C and D)** The 9 mg/kg group showing some bi-nucleated hepatocytes (arrow head), dilated portal vein (PV) and inflammatory leucocytes. Severe congestion of hepatic tissue (blue arrow) and CVs, vacuolation of some hepatocytes (orange head arrow), and pyknotic nuclei (black arrow) were observed**. (E and F)** The 18 mg/kg group showing central vein with thick fibers (F), many bi-nucleated cells (arrowhead), and hemosiderin (white arrow). A degenerated area (D), and hydropic degeneration (HD), fibrosis (F), and apoptotic areas (*) were widely observed. **(H and I)** The 27.5 mg/kg and **(J and K)** 55 mg/kg groups show ballooning of hepatocytes, necrosis (N), HD, few pyknotic nuclei, karyolytic nuclei (orange arrow), and congested portal vein (PV) (H and E stain, scale bar = 50 μm). **(B)** Liver histopathology scores by Heijnen’s methods, different signs in column are significantly different (P < 0.001).

The microscopic examinations of the liver female and male wild rats dosed with warfarin are shown in Fig. 7. The architecture of the liver in the control group appeared normal (Fig. 7A and G). Group III (9 mg/kg) had an abnormality in the hepatic architecture, with karyolitic and some bi-nucleated cells, hemolysis that filled the portal vein and enlarged portal tract, a small number of Kupffer cells, and many inflammatory leucocytes (Fig. 7C). Some of the hepatocytes were vacuolated and others had pyknotic nuclei, hemorrhage of interlobular areas, and congested central veins (Fig. 7D). The 18 mg/kg group had acute loss of the normal organization of the hepatic plates, the central vein exhibited significant fibrosis, a widely hydropic area, and hemosiderin deposition (Fig. 7E). Proliferation of bile duct, dilated central veins, and apoptotic tissues and inflammatory cells were also observed **(**Fig. 7F**).** The 27.5 mg/kg group had loss of blood sinusoids, the appearance of hydropic degeneration, and numerous cells with pyknotic nuclei (Fig. 7H), and cells with bi-nucleated, karyolytic nuclei (Fig. 7I). The 55 mg/kg group had severe destruction of hepatic tissue with extensive areas of fibrosis and hepatocytes with darkly stained nuclei (Fig. 7J). Moderate necrosis, inflammation and congested portal vein and few amount of hemosiderin were widely apparent (Fig. 7K). Determination of the toxicity of warfarin using Heijnenʼs score of the different groups showed significant increases of 674, 1017.5, 819.7, and 937.3%, respectively, compared with the control group (Fig. 7B).

### 3.10 Liver fibrosis and morphometric analysis

**Fig. 8:**
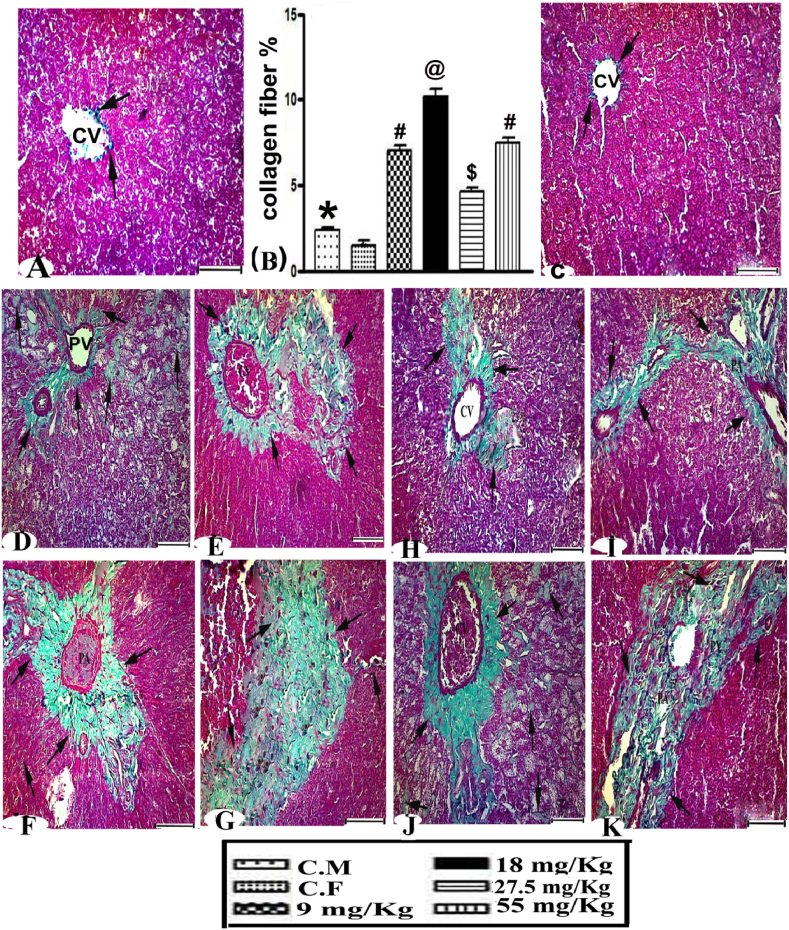
**(A)** Masson’s trichrome stained liver tissue of control and **(D and E)** 9 mg/kg and **(H and I)** 27.5 mg/kg of warfarin revealed moderate amounts of collagen fiber (arrows). **(F and G)** The 18 mg/kg and **(J and K)** 55 mg/kg groups had a huge amount of fiber (arrows) among the hepatocytes, the portal vein (PV), and the central vein (CV) (scale bar = 50 μm). **(B)** The percentage of liver fibrosis and values of different signs are significantly different (P < 0.001).

Tissue sections were additionally stained with Masson’s stain to estimate the liver fibrosis and facilitate morphometric analysis (Fig. 8). Control female and male wild rats had a normal amount of collagenous fibers around the central veins (Fig. 8 A and C). The 9 and 27.2 mg/kg groups showed the beginnings of the emergence of fiber hyperplasia (collagenous fibers) around the portal vein, the bile duct, and in the arteriovenous regions (Fig. 8D and I). A significantly increased number of fibers was seen around the congested central veins and through the degenerated hepatocytes (Fig. 8E and H). Groups IV and VI (18 and 55 mg/kg) had a huge amount of fiber hyperplasia, leading to collagen deposits around the portal areas and central vein (Fig. 8F and J) and causing bridging of fibers between the hepatocytes and sinusoids (Fig. 8G and K). Morphometric analysis of liver fibrosis in the 9, 18, 27.5 and55 mg/kg groups demonstrated significant increases of 381.33, 598.71, 96.03, and 216.73%, respectively, compared with control rats (Fig. 8B).

## 4 Discussion

In the current study we discovered that oral intake of low and high doses of warfarin for 18 days resulted in the stimulation of ROS and oxidative stress in male and female wild rats. The most effective technique to get rid of rats is with anticoagulant rodenticides like warfarin (4-OH coumarin) [40,41]. We were able to pinpoint the negative effects of warfarin therapy—including bleeding—that caused wild rats to die, as assured by Ref. [42]**,** and identified the LD50 in female and male rats. Regardless of the animal species, or the quantity of warfarin, all investigators have recorded hemorrhages in the eye, skin, muscles, viscera, and lungs of animals [43].

The current findings indicate that warfarin causes damage to liver cells by lowering the levels of NRF2, an oxidative stress marker, and increasing cleaved caspase-3, an apoptotic cell death marker. This damage leads to warfarin's pro-hemorrhagic effects and a shift in the defense system, and is the first explanation of the toxicity of warfarin toward rats. Warfarin may affect the NRF2 pathway to prevent Nrf2 dissociating from Kelch-like ECH-associated protein 1 (Keap1), or cause Nrf2 mutations, so that it is not translocated into the nucleus during oxidative stress, and cannot bind to antioxidant response elements or activate antioxidant protection enzymes such as catalase, glutathione peroxidase, and SOD. The absence of Nrf2 results in an increase in oxidative/nitrosative stress [44,45], and the lack of Nrf2 can exacerbate NF–B activity, resulting in increased inflammatory cytokine release in kidney injury in rodents [46].

Nrf2 has been shown, in a variety of studies, to be an inhibitor of apoptosis, including Fas-induced apoptosis, and nitric oxide-induced apoptosis [47]. As a result, caspase-mediated cell death is a more fitting summary of our findings [48]. The findings of this study suggest that apoptosis may play a role in the pathophysiology of cell death following a warfarin-induced liver hemorrhage. Given warfarin's cytotoxicity in terms of oxidative stress, it may cause apoptosis in the leukemia cell lines K562 and HL-60 because of a rise in cytochrome C release, or this effect may be due to superoxide radicals (O2) formed by reactive oxygen species [49]. Warfarin metabolism is affected by genetic polymorphism, particularly CYP2 mutations, which leading to poisoning [5,50]. Our results revealed a moderate rise in CYP2C9 after warfarin intake, but this increase did not prevent bleeding, and this finding was synchronized with the previous CYP2C9 (primary metabolizer of (S)-warfarin) pathway [51]. Overdosing on warfarin causes a CYP2C9 polymorphism, which causes bleeding [52,53]. The regulation of cytochrome P450 genes such as CYP2A5 and CYP2C9 in mice appears to be Nrf2-dependent [54]. This observation supported our previous findings, which showed a close connection between NRF2 and CYP2C9.

In the current research, there is evidence of a relationship between a decrease in serum albumin levels and high bleeding risk due to exposure to warfarin. Hypoalbuminemia is likely to increase the free fraction of warfarin and increase the risk of bleeding, because warfarin is mainly bound to serum albumin [55]. We found that warfarin decreased antitrypsin, resulting in excessive inflammatory activation, including an increase total white blood cells and neutrophils, and a decrease in RBC, lymphocytes, and hemoglobin, and stimulated liver fibrosis. This finding indicated that antitrypsin and the systemic modulatory response are linked [56]. Activation of hepatic stellate cells after Alpha-1 antitrypsin deficiency may be due to increased levels of pro-fibrogenic molecules such as transforming growth factor beta 1 in liver fibrosis of mice [57]. This mechanism is in keeping with our findings on the function of antitrypsin in fibrosis and apoptosis. All of these fibrosis-promoting conditions have increased iron rates. Excess iron (Fe) is harmful, and leads to the production of noxious reactive oxygen species in cells and tissues. In our study, warfarin led to an increase in serum transferrin, and the accumulation of hemosiderin. Similar findings were seen in rat HSCs, in which iron increased cell proliferation and collagen synthesis. Hemosiderin is frequently generated after bleeding, according to Ref. [58], as red blood cells pass away and release hemoglobin into the extracellular environment [59].

Reactive oxygen species-induced malondialdehyde, a producer of lipid peroxidation in membranes of cellular organelles, increased the expression of COL-1a1 and TGF- in iron-loaded rats [60]. Previous research has shown that hepatic deposition of collagen fibers is linked to serum concentrations of liver and renal function elevated [61]. All of these findings support our conclusion that the experimental model is suitable for discovering new biomarkers of liver fibrosis. The reduction in leukocyte counts in rat strains was due to a decline in lymphocyte counts in the peripheral blood of rats which ingested warfarin interperitoneally, due to leukocyte migration to hemorrhage foci. They are probably linked to intestinal injury in warfarin-treated rats during the blood response to tissue injury [62]. Differential white blood cell responsiveness to oral warfarin may be a response to pro-inflammatory cytokines and the expression of TNF-α and its effects on bone marrow [63].

Our results revealed that warfarin caused severe histopathological and antioxidants changes, oxidative stress biomarkers, and biochemical parameters in plasma. Many studies support our findings that transaminase elevation and warfarin use are related, as ALT and AST levels increase in tandem with a rise in bilirubin level [64]. In liver and duodenal tissue injury in humans and rats oxidative activities, pro-inflammatory cytokine responses, increases in plasma AST, and proteinuria were observed, as a result of necrosis and high warfarin intake [65,66]. In another interpretation, it was found that elevation of the AST and ALT levels released into the bloodstream caused liver damage due to the permeability of the hepatocellular membrane under pathological conditions [67].

Variance in CAT and SOD activity may occur as a result of the need to activate protective mechanisms to scavenge reactive oxygen species produced in plasma [68]. According to Ref. [69], there is a strong positive link between the deposition of fibronectin, collagen I, and a reduction in NO, and liver damage. Also, it has been suggested that Vitamin K deficiency has been linked to anticoagulant instability and a reduction in the activity of endogenous antioxidant enzymes [70] this observation supports our results. Deficiency of GSH, cell death, and human hepatic congestion are brought on due to the slow and gradual accumulation of ROS within the cells, resulting in oxidative stress [71].

## 5 Conclusion

Our findings indicate that the oral intake of warfarin increased oxidative stress, leading to toxicity in the livers of wild rats (female and male), by the following mechanisms: A) the regulation of CYP2C9 appears to be dependent on NRf2 and the level of P450, and affects the amount of bleeding. B) A decrease in NRf2 is considered to be a non-defense system against oxidative stress, decreasing the endogenous antioxidant defense system and accelerating hemorrhage. C) Free hemoglobin and decreased serum albumin and antitrypsin activate cleaved caspases-3, inducing apoptosis and fibrosis after liver hemorrhage and bleeding. D) Warfarin increases serum transferrin and lipid peroxidation, which are pro-fibrogenic stimuli, by elevating iron levels and hemosiderin, promoting fibrosis and the pathogenesis of acute liver injury**.**

## Ethical approval

I confirm, the study of our manuscript is reported in accordance with ARRIVE guidelines.

## Additional information

No additional information is available for this paper.

## Data availability statement

Data will be made available on request.

## Funding

No fund. This research did not receive any specific grant from funding agencies in the public, commercial, or not-for-profit sectors.

## CRediT authorship contribution statement

**Mona M. Atia:** Writing – review & editing, Writing – original draft, Visualization, Validation, Supervision, Software, Resources, Methodology, Formal analysis, Data curation, Conceptualization. **Heba Allah Ahmed Mahmoud:** Writing – original draft, Resources, Methodology, Formal analysis, Data curation, Conceptualization. **Magdy Wilson:** Writing – review & editing, Supervision, Methodology, Conceptualization. **Elham A. Abd-Allah:** Writing – review & editing, Writing – original draft, Visualization, Validation, Supervision, Resources, Project administration, Methodology, Formal analysis, Data curation, Conceptualization.

## Declaration of competing interest

The authors declare the following financial interests/personal relationships which may be considered as potential competing interests:Mona M Atia reports administrative support was provided by Assiut University. If there are other authors, they declare that they have no known competing financial interests or personal relationships that could have appeared to influence the work reported in this paper.

## List of abbreviation

**CYP2C9**: Cytochrome P450
**COL1A1**: Collagen 1 alpha 1
**NRF2**: Nuclear factor erythroid 2-related factor 2
**AST**: Aspartate aminotransferase
**ALT**: Alanine aminotransferase
**ALP**: Alkaline phosphatase
**LPO**: Lipid peroxidation
**GSH**: Reduced glutathione
**CAT**: Catalase
**SOD**: Superoxide dismutase
**NO**: Nitric oxide.
